# The Effect of Intraoperative Cerebral Oximetry Monitoring on Postoperative Cognitive Dysfunction and ICU Stay in Adult Patients Undergoing Cardiac Surgery: An Updated Systematic Review and Meta-Analysis

**DOI:** 10.3389/fcvm.2021.814313

**Published:** 2022-02-01

**Authors:** Li-Juan Tian, Su Yuan, Cheng-Hui Zhou, Fu-Xia Yan

**Affiliations:** Department of Anesthesiology, State Key Laboratory of Cardiovascular Disease, National Center for Cardiovascular Diseases, Fuwai Hospital, Chinese Academy of Medical Sciences and Peking Union Medical College, Beijing, China

**Keywords:** cardiac surgery, cardiopulmonary bypass, postoperative delirium, regional cerebral oxygen saturation, postoperative cognitive decline

## Abstract

**Aim:**

Determining whether intraoperative cerebral oximetry monitoring-guided intervention reduces the risk of postoperative cognitive dysfunction remains controversial. The objective of this study was to conduct an up-to-date meta-analysis to comprehensively assess the effects of regional cerebral oxygen saturation (rSO_2_) monitoring-guided intervention on cognitive outcomes after cardiac surgery.

**Methods:**

PubMed, EMBASE, Ovid, and Cochrane Library databases were systematically searched using the related keywords for cardiac surgical randomized-controlled trials (RCTs) published from their inception to July 31, 2021. The primary outcome was postoperative delirium (POD). The secondary outcomes were postoperative cognitive decline (POCD) and other major postoperative outcomes. The odds ratio (OR) or weighted mean differences (WMDs) with 95% confidence interval (CI) were used to pool the data. The random-effect model was used for the potential clinical inconsistency. We performed meta-regression and subgroup analyses to assess the possible influence of rSO_2_ monitoring-guided intervention on clinical outcomes.

**Results:**

In total, 12 RCTs with 1,868 cardiac surgical patients were included. Compared with controls, the incidences of POD (*n* = 6 trials; OR, 0.28; 95% CI, 0.09–0.84; *p* = 0.02; *I*^2^ = 81%) and POCD (*n* = 5 trials; OR, 0.38; 95% CI, 0.16–0.93; *p* = 0.03; *I*^2^ = 78%) were significantly lower in the intervention group. Cerebral oximetry desaturation also showed a positive association with the incidence of POD (*n* = 5 trials; OR, 2.02; 95% CI, 1.25–3.24; *p* = 0.004; *I*^2^ = 81%). The duration of intensive care unit (ICU) stay was markedly shorter in the intervention group than in the control group (*n* = 10 trials; WMD, −0.22 days; 95% CI, −0.44 to −0.00; *p* = 0.05; *I*^2^ = 74%). Univariate meta-regression analyses showed that the major sources of heterogeneity were age (*p* = 0.03), body mass index (BMI, *p* = 0.05), and the proportion of congenital heart disease (CHD, *p* = 0.02) for POD, age (*p* = 0.04) for POCD, diabetes mellitus (DM, *p* = 0.07), cerebrovascular accident (CVA, *p* = 0.02), and chronic obstructive pulmonary disease (COPD, *p* = 0.09) for ICU stay. Subsequent subgroup analyses also confirmed these results.

**Conclusion:**

Available evidence from the present study suggests that an intraoperative cerebral oximetry desaturation is associated with an increased POD risk, and the rSO_2_ monitoring-guided intervention is correlated with a lower risk of POD and POCD, and a shorter ICU stay in adults undergoing cardiac surgery. These clinical benefits may be limited in patients with older age, diabetes status, high BMI, non-CHD, non-COPD, or a previous cardiovascular accident.

**Systematic Review Registration:** [PROSPREO], identifier: [CRD42021252654].

## Introduction

Transient cognitive dysfunction following cardiac surgery with cardiopulmonary bypass (CPB) is a common and clinically important complication (1). Postoperative delirium (POD) is the most severe presentation of neurocognitive disorders with a rate of up to 50% of cardiac surgical patients (2), which contributes to a prolonged hospital stay, long-term cognitive impairment, and increased morbidity and mortality (1, 3–7). Some previous studies showed that patients were susceptible to neurocognitive disorders from hypoperfusion and microemboli resulting in impaired cerebrovascular autoregulation during cardiac surgery undergoing CPB (8). In a cohort study concerning cardiac surgery, intraoperative post-ischemia cerebral hyper-oxygenation has been shown to be strongly associated with an increased risk of postoperative cognitive dysfunction (9). Near-infrared spectroscopy (NIRS) possesses the potential of non-invasively evaluating the oxygen supply/demand balance in frontal brain tissue and providing real-time regional cerebral oxygen saturation (rSO_2_) even during non-pulsatile perfusion, and intraoperative decreased rSO_2_ may indicate a clinically relevant association with cognitive dysfunction (10–12). Additionally, there are very few literature studies supporting that anesthetic practice based on optimizing cerebral oxygenation during cardiac surgery leads to improved postoperative outcomes (13, 14). However, this conclusion was still controversial as some recently randomized-controlled trials (RCTs) have demonstrated that NIRS-guided intervention has no effect on the reduction of neurocognitive disorders after cardiac and non-cardiac surgeries (15, 16). Two prospective randomized studies, including high-risk patients conducted by Lei et al. and Deschamps et al., found that NIRS-guided intervention could attenuate the decreases of rSO_2_ in cardiac surgery but did not affect the incidence of POD (17, 18).

Therefore, the effect of intraoperative NIRS-guided intervention on postoperative cognitive dysfunction in cardiac surgical patients with CPB remains unclear. An up-to-date systematic review and meta-analysis aim to comprehensively evaluate the effects of intraoperative anesthetic practice based on cerebral oximetry monitoring on delirium and cognitive outcomes after cardiac surgery.

## Methods

This study followed the methodology outlined in the Cochrane Handbook for Systematic Reviews of Interventions Version 6.0 (19). We explained it in accordance with the Preferred Reporting Items for Systematic Reviews and Meta-Analyses Protocols (PRISMA) statement. This protocol has been registered on the International Prospective Systematic Reviews Registry database (PROSPERO 2021: CRD42021252654).

### Search Strategy

PubMed, EMBASE, Ovid, and Cochrane Library databases were searched for English articles published from their inception to July 31, 2021, for RCTs evaluating the effects of intraoperative anesthetic practice followed cerebral oximetry monitoring on post-cardiac surgery cognitive outcomes. The related ongoing or completed studies on ClinicalTrials.gov were also searched, and the references of the identified studies were also reviewed to identify further relevant studies. The related searching words were as follows: (Postoperative delirium) OR (Postoperative Cognitive Dysfunctions) AND [(cardiac surgery) OR (cardiopulmonary bypass) OR (coronary artery bypass surgery) OR (valve surgery) OR (aortic surgery) OR (congenital heart disease)] AND (intraoperative cerebral oximetry) AND (randomized controlled trial OR controlled clinical trial OR randomly OR trial) in the title/abstract. In addition, we manually searched the references of the identified studies.

### Selection Criteria

The study selection criteria were as follows: (1) Population: populations of interest were adult patients undergoing cardiac surgery. Studies concerning children, infants, or newborns were excluded. (2) Intervention: The intervention group was NIRS-guided therapy. (3) Comparator: The intervention group vs. the control group. (4) Outcome: The incidence of POD. (5) Study design: Only included RCTs to ensure that the combined results were of good quality, and excluded the studies that could not provide effective analysis data.

### Interventions

Thresholds for intervention were generally below 70–90% baseline rSO_2_ or below 50–60% absolute rSO_2_. Intraoperative cerebral oximetry-guided therapy was considered as the primary intervention and was triggered by the evidence of cerebral oxygen desaturation. Specific interventions included the fluid supplement and/or vasoactive drugs for hypotension, changes in ventilatory parameters to optimize the partial pressure arterial oxygen and carbon dioxide, or blood transfusion.

### Outcomes and Definitions

The primary outcome was the incidence of POD as defined in the individual studies. The secondary outcomes were postoperative cognitive decline (POCD) and intensive care unit (ICU) stay. Additional outcomes included mechanical ventilation duration, hospital length of stay (LOS), as well as the incidence of myocardial infarction (MI), acute kidney injury (AKI), surgical site infection, cerebrovascular accident (CVA), and hospital mortality.

### Data Collection and Quality Assessment

Two researchers (LJT and CHZ) independently extracted the following study design and patient characteristics: research title, author's name, the year of publication, the journal of included studies, research area (hospital or research institute), total number of patients, the number of patients in the two groups, gender, age, the type of surgical procedure, and the data regarding outcomes of interest in both groups.

The two researchers evaluated the quality of the included studies in accordance with the quality assessment section of the Cochrane handbook for systematic reviews of interventions 6.0. Two researchers also assessed selection bias, blind bias, incomplete outcome data bias, selective reporting bias, and other biases for each included study (20). Disagreements were resolved through a discussion during the process of data abstraction. The quality of the study was categorized as low-, medium-, or high-risk accordingly. In addition, the baseline characteristics and comorbidity were compared between the intervention and control groups in each study for patients.

### Statistical Analysis

All the data were analyzed by Review Manager 5.4 (Cochrane Collaboration, Oxford, UK), Stata 12.0 (Stata Corporation, College Station, TX, USA), and Trial Sequential Analysis 0.9 Beta (Copenhagen Trial Unit, Copenhagen, Denmark). The odds ratios (ORs) with 95% confidence intervals (CIs) were estimated for dichotomous data, and weighted mean differences (WMDs) with 95% CIs for continuous data, respectively. In addition, we converted the data expressed as the median and interquartile range (IQR), to mean and SD by the formulas of Luo and Wan (21). The random-effect model was used to pool the data for the consideration of methodological and clinical heterogeneity. *Q*-test (*p* < 0.1 denoted statistically significant heterogeneity) and *I*^2^ statistics (*I*^2^ > 50% was considered as the presence of significant heterogeneity) were used to evaluate the heterogeneity of this study. Meta-regression (*p* < 0.1) and subgroup analyses were conducted for positive results to explore the potential sources of heterogeneity between rSO_2_ monitoring-guided intervention and clinical outcomes. To reduce the possibility of overfitting in the regression model, at least four studies or substudies were set for the identification of every one influential factor (22). Publication bias was evaluated with the Egger's and Begg's tests (23). We performed the trial sequential analyses (TSAs) of POD, POCD, and ICU stay based on the data from our pooled analysis (OR and the incidence of POD or POCD; WMD and variance) to calculate the required sample size for the statistical power. *P* < 0.05 was set as statistically significant.

## Results

### Literature Search, Study Characteristics, and Quality Assessment

**Figure 1:**
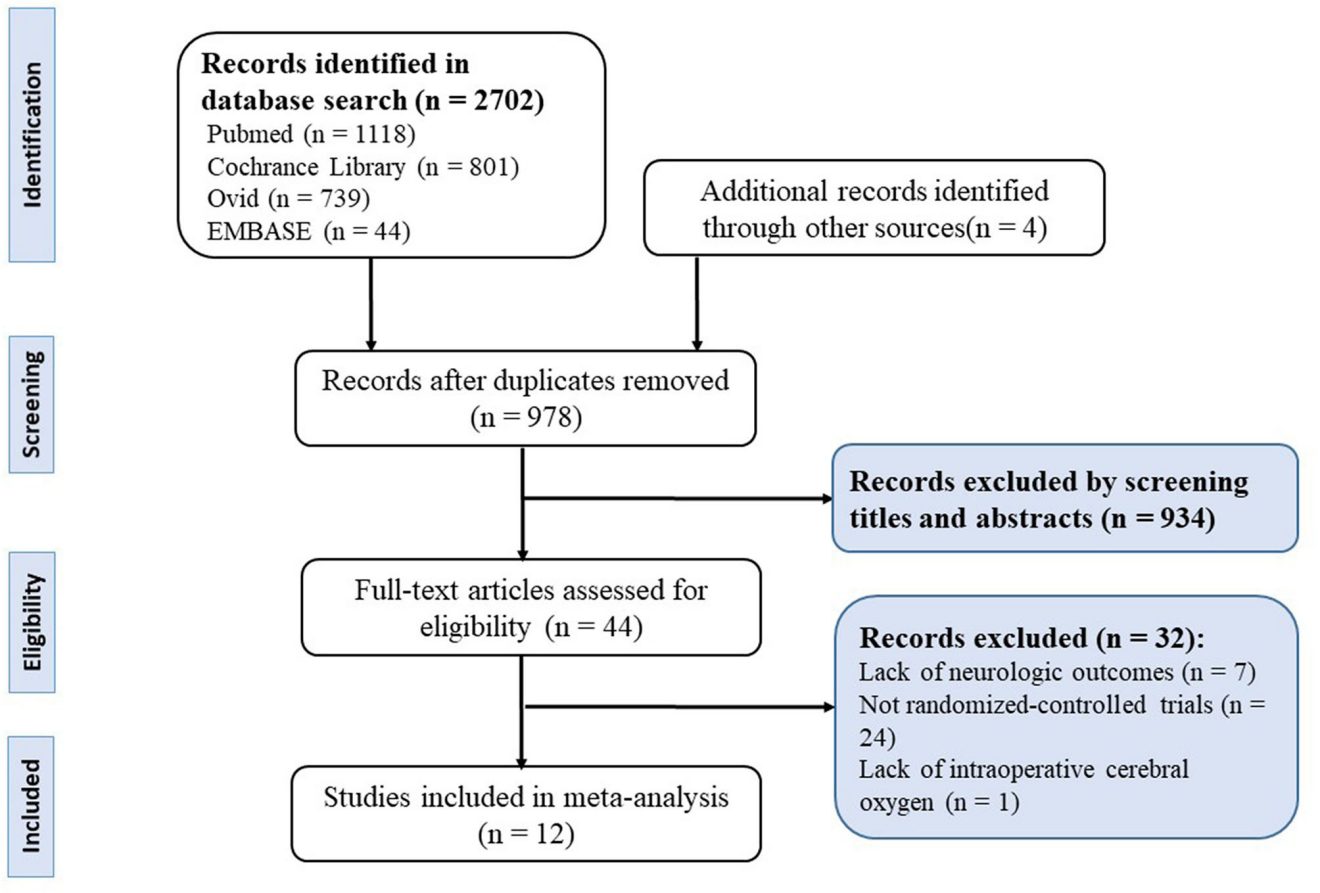
Preferred reporting items for systematic reviews and meta-analyses (PRISMA) flowchart of selection.

As depicted in the flow chart (Figure 1), our initial search yielded 2,702 records. A total of 2,662 trials were excluded by being duplicated and reviewing the titles and abstracts. Twenty-four of the trials were excluded for a non-RCT design. Seven trials were terminated without neurologic outcomes. One trial was excluded due to the exploration of the relationship between postoperative cerebral oxygen and delirium. In total, 12 RCTs with 1,868 adult cardiac surgical patients were included in this meta-analysis (16–18, 24–32).

### Study Patient and Intervention Characteristics

The study characteristics are presented in Tables 1, 2. The screening included 1,868 patients undergoing cardiac surgery (on-pump coronary artery bypass graft (CABG), valve surgery, and CABG combined valve surgery) for meta-analysis, including 934 patients allocated into the intervention group and 934 in the control group. The definition criteria of cerebral oximetry desaturation in each study vary from below 70–90% of baseline rSO_2_ or below 50–60% absolute rSO_2_. The interventions to correct cerebral oxygen desaturation included the optimization of mechanical ventilation strategy, vasopressor delivery, fluid administration, and blood transfusion.

**Table 1:** Baseline characteristics of included studies for meta-analysis.

**References**	**Sample size**		**Age (y)**		**Sex (M/F)**		**Surgery**	**Intervention**	**Outcome**
	**Intervention**	**Control**	**Intervention**	**Control**	**Intervention**	**Control**			
Uysal et al. (16)	59	66	57 ± 11	58 ± 12	40/19	46/20	Cardiac surgery	rSO_2_ below 60% for 1 min or longer	POD, ICU stay, hospital LOS, transfusion requirement and SOFA on ICU admission
Deschamps et al. (17)	102	99	69 ± 12.6	72 ± 9.4	74/28	71/28	Cardiac surgery	rSO_2_ decreased by 10% of baseline for a duration exceeding 15 s	POD, ICU stay, hospital LOS, MI, transfusion requirement, infection and MOMM
Lei et al. (18)	123	126	74.2 ± 6.5	72.9 ± 6.3	88/35	88/38	Cardiac surgery	rSO_2_ below 75% of the baseline value for 1 min or longer	POD, ICU stay, hospital LOS, MI, transfusion requirement, infection and mortality
Murkin et al. (24)	100	100	61.8 ± 9.3	61.8 ± 10.3	87/13	88/12	CABG	rSO_2_ values at or above 75% baseline value	ICU stay, hospital LOS, wound infection and MI
Slater et al. (25)	125	115	64.33 ± 10.2	65.19 ± 9.7	104/21	97/22	CABG	rSO_2_ drop more than 20% baseline value	POCD and hospital LOS
Vretzakis et al. (26)	75	75	67.3 ± 8.5	65.9 ± 9.5	63/12	60/15	Cardiac surgery	rSO_2_ <60% or decreased by 20% of baseline	ICU stay, hospital LOS and transfusion requirement
Deschamps et al. (27)	23	25	71.1 ± 7.9	70.2 ± 9.2	19/4	14/11	High-risk cardiac surgery	rSO_2_ decreased by 20% of baseline for a duration exceeding 15 sec	ICU stay, hospital LOS, and transfusion requirement
Mohandas et al. (28)	50	50	34.6 ± 16.3	38.1 ± 15.8	30/20	28/22	Open heart surgery	rSO_2_ below 85% of the baseline or below 50% for 1 min or longer	POCD (postoperative 1 week and 3 months)
Colak et al. (29)	94	96	61.9 ± 7.1	63.4 ± 8.8	75/19	73/23	CABG	rSO_2_ below 80% of the baseline or below 50%	POD POCD, ICU stay, hospital LOS, MI, transfusion requirement and infection rate
Kara et al. (30)	43	36	59.1 ± 9.4	61.2 ± 10.3	33/10	29/7	CABG	rSO_2_ below 80% of the baseline	POCD, ICU stay and hospital LOS
Rogers et al. (31)	98	106	65.9 ± 22.7	70 ± 19.7	66/32	74/32	Open valve or combined CABG and valve surgery	rSO_2_ below 70% of the baseline or below 50%	ICU stay, hospital LOS, MI, transfusion requirement, infection and mortality
Kunst et al. (32)	42	40	71.6 ± 5	72 ± 4.3	33/9	34/6	CABG	rSO_2_ below 85% of the baseline or below 50%	POD and POCD, ICU stay and hospital LOS

*CABG, coronary artery bypass graft; POD, postoperative delirium; POCD, postoperative cognitive decline; rSO_2_, regional cerebral oxygen saturations; MI, myocardial infarction; ICU, intensive care unit; LOS, length of stay; SOFA, sequential Organ failure assessment; MOMM, major organ morbidity and mortality*.

**Table 2:** Baseline characteristics, medical conditions, and perioperative data of included studies for meta-analysis.

**References**	**Age (Year)**	**Male (%)**	**BMI**	**Pre-MI (%)**	**DM (%)**	**HT (%)**	**CVA (%)**	**COPD (%)**	**CRF (%)**	**CPB duration (min)**	**Euroscore**	**Baseline LVEF (%)**	**CABG (%)**	**CHD (%)**	**Valve surgery (%)**	**Complex surgery (%)**
Uysal et al. (16)	57.5	68.8	26.8	NA	NA	NA	NA	NA	NA	131.1	2.5	60.0	5.6	0	67.2	27.2
Deschamps et al. (17)	71.0	72.1	NA	4.5	29.4	79.6	NA	9.9	13.9	135.9	5.3	NA	NA	0	28.2	70.1
Lei et al. (18)	73.5	70.7	28.1	12.4	27.7	76.7	14.5	12.9	NA	111.0	NA	NA	55.8	0	NA	43.8
Murkin et al. (24)	61.8	87.5	29.6	5	28.5	NA	7.0	17.5	8.5	88.2	NA	NA	100	0	0	0
Slater et al. (25)	64.7	83.5	NA	16.0	32.9	78.3	7.5	5.6	4.4	65.6	NA	51.1	100	0	0	0
Vretzakis et al. (26)	66.6	82.0	27.8	54.7	24	80.6	NA	21.3	NA	91.3	NA	47.8	80.7	1.3	10.0	8.0
Deschamps et al. (27)	70.6	68.8	NA	NA	NA	NA	NA	NA	NA	116.6	NA	56.3	19.0	0	23.8	50.2
Mohandas et al. (28)	36.3	58.0	20.7	NA	NA	NA	NA	NA	NA	88.7	NA	NA	0	20.0	74.0	6.0
Colak et al. (29)	62.7	78.0	NA	11.6	33.7	90.5	NA	NA	1.6	90.0	2.3	56	100	0	0	0
Kara et al. (30)	60.1	78.5	NA	NA	30.4	74.7	12.7	15.2	1.3	78.1	NA	54.0	100	0	0	0
Rogers et al. (31)	68.0	69.0	27.6	10.0	9.0	NA	8.0	NA	NA	108.8	5.0	NA	0	0	77.0	23.0
Kunst et al. (32)	71.8	81.7	26.7	NA	31.7	92.7	14.6	11.0	NA	81.1	4.4	NA	100	0	0	0

*BMI, body mass index; MI, myocardial infarction; DM, diabetes mellitus; HT, hypertension; CVA, cerebrovascular accident; COPD, chronic obstructive pulmonary disease; CRF, chronic renal failure CPB, cardiopulmonary bypass; Euroscore, European system for cardiac operative risk evaluation; EF, ejection fraction; CABG, coronary artery bypass graft; CHD, congenital heart disease; NA, not available*.

### Quality Assessment

**Figure 2:**
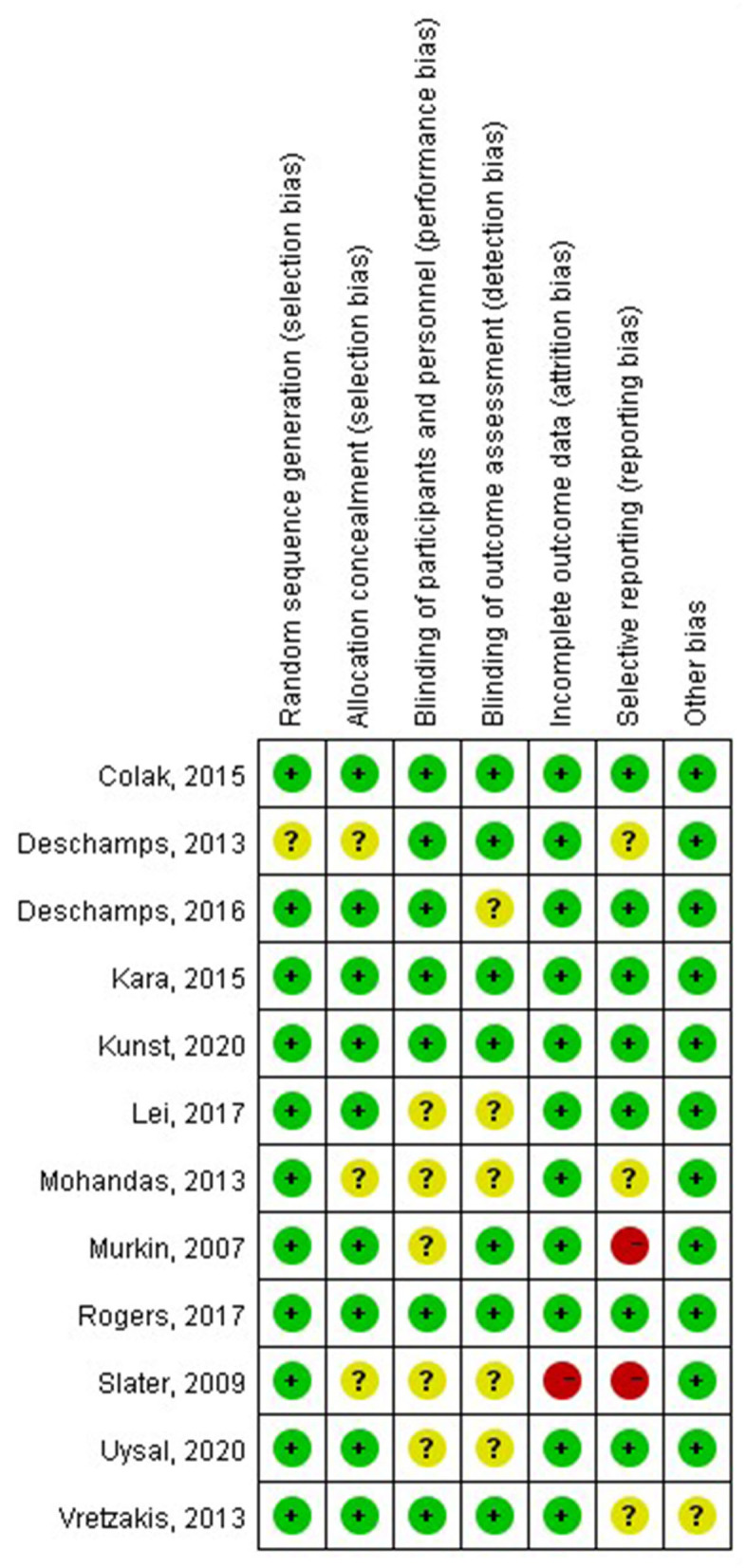
Risk of bias summary of review authors' judgments about each risk of bias item for each included study.

**Figure 3:**
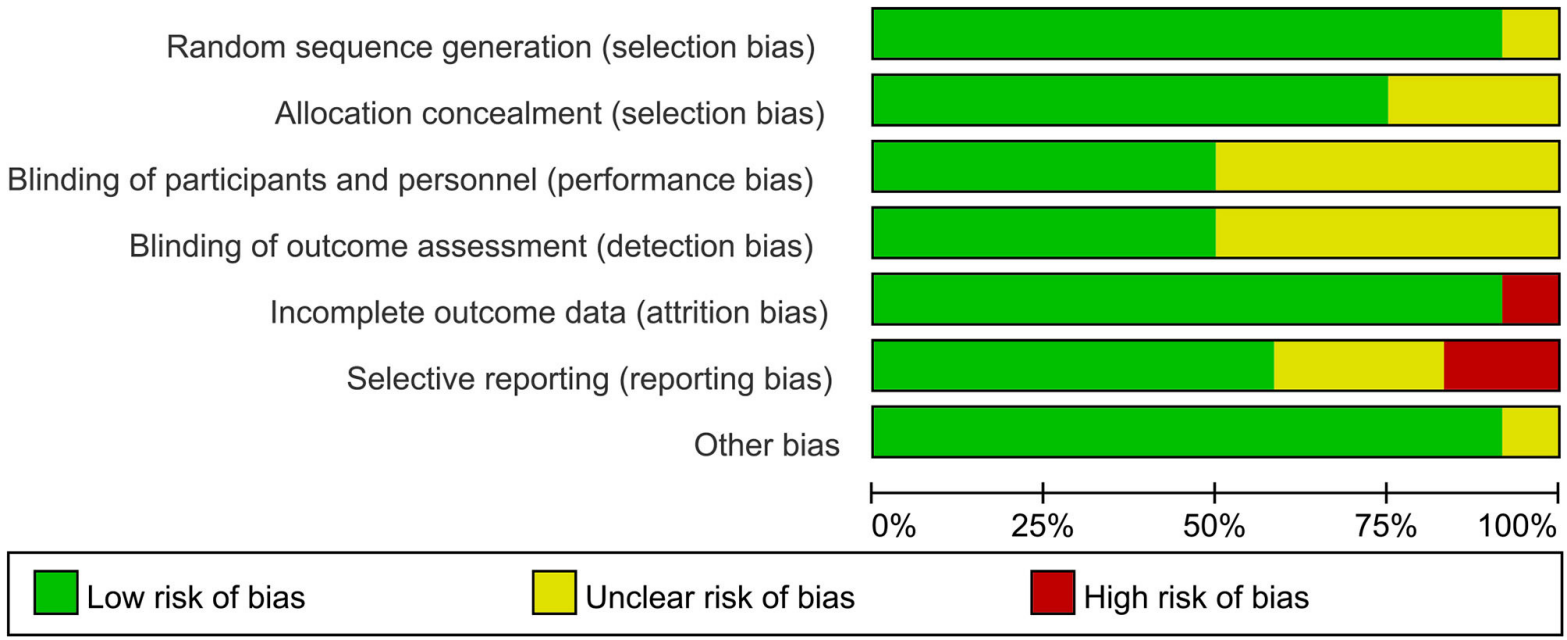
Risk of bias graph of review authors' judgments about each risk of bias item presented as the percentage across all included studies.

Six studies with an unclear risk of bias were due to the unclear blinding assessments or participants and personnel (16, 17, 26–28). Two, trials did not describe the study design in detail and were not clear about the selective reporting bias (24, 25), almost all the included RCTs were assessed as low bias risk, indicating that they were of good quality (Figures 2, 3).

### Primary Outcomes

**Figure 4:**
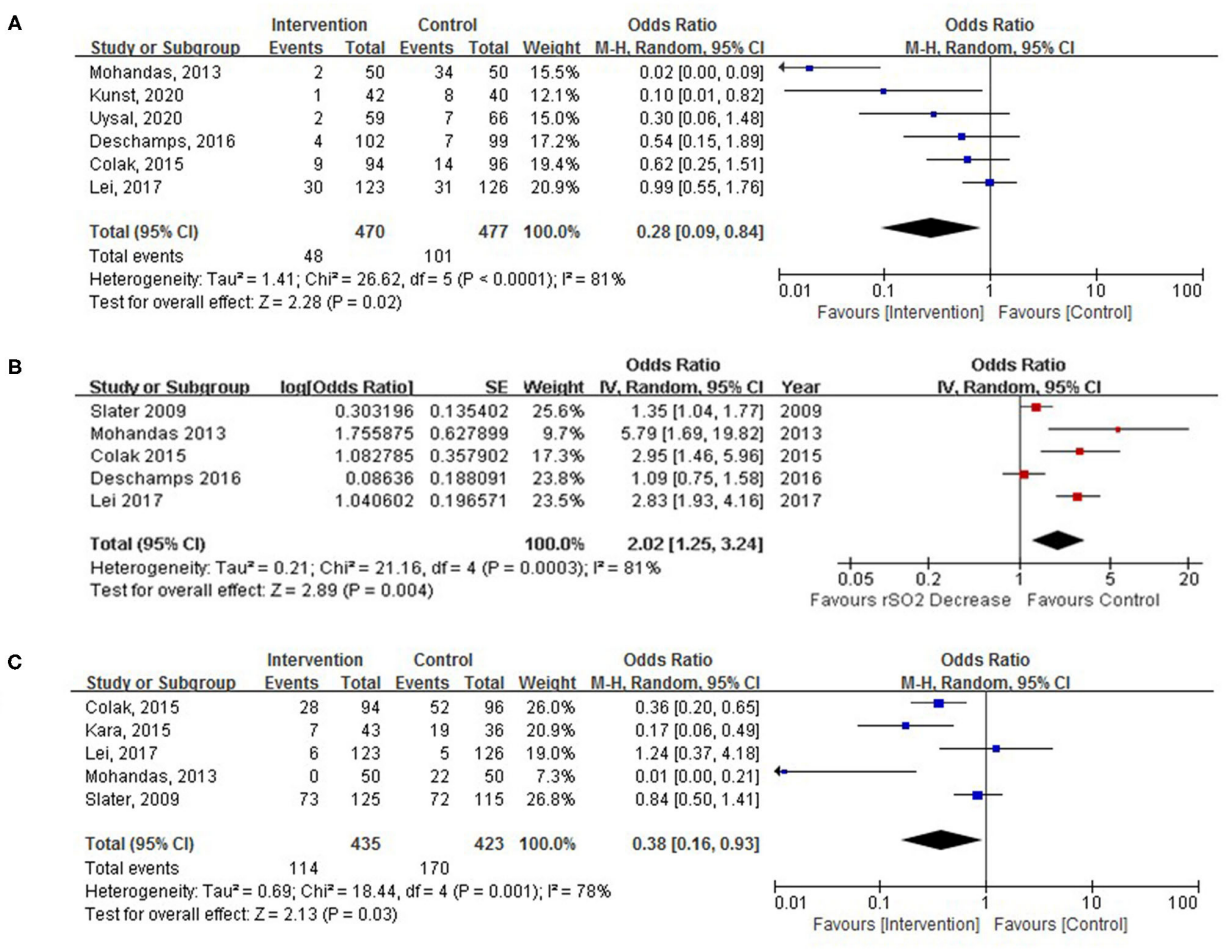
**(A)** Pooled effect of cerebral oximetry monitoring-guided intervention on the incidence of postoperative delirium (POD). **(B)** The pooled incidence of POD with cerebral oximetry desaturation. **(C)** The pooled effect of cerebral oximetry monitoring-guided intervention on the incidence of postoperative cognitive decline (POCD).

Six RCTs with 826 patients were assessed for POD with an overall incidence of 18.0% (intervention, 11.9%; control, 23.8%). There was a significant decrease in the incidence of POD in the intervention group relative to the control group (Figure 4A; OR, 0.28; 95% CI, 0.09–0.84; *p* = 0.02; *I*^2^ = 81%).

There was an increased occurrence of POD with cerebral oximetry desaturation defined by both relative (below 70–90% baseline rSO_2_) or absolute (below 50–60% absolute rSO_2_) thresholds (Figure 4B; OR, 2.02; 95% CI, 1.25–3.24; *p* = 0.004; *I*^2^ = 81%) in five RCTs including 859 patients undergoing cardiac surgery.

### Secondary Outcomes

The POCD was reported in 858 study participants, and the overall incidence was 31.1% (intervention, 114/435; control, 170/423). POCD was reported in five trials, there was a significant reduction in POCD after cardiac surgery in the intervention group (Figure 4C; OR, 0.38; 95% CI, 0.16–0.93; *p* = 0.03; *I*^2^ = 78%).

**Figure 5:**
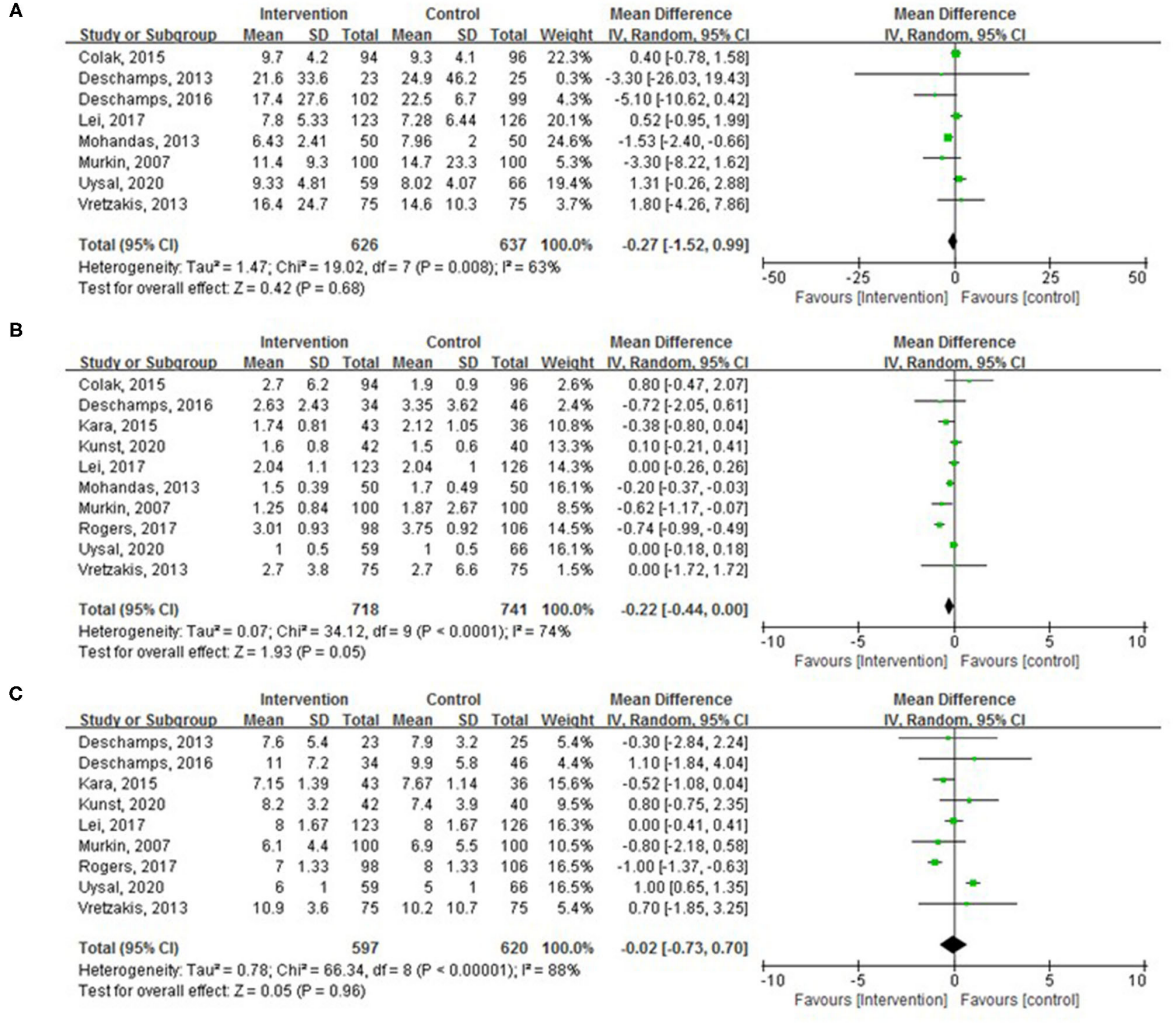
Pooled effect of cerebral oximetry monitoring guided intervention on **(A)** mechanical ventilation duration, **(B)** intensive care unit (ICU) length of stay (LOS), and **(C)** length of postoperative hospital stay.

The mechanical ventilation duration was explored in eight trials with 1,263 patients without a statistically significant difference between the two groups (Figure 5A; WMD, −0.27 h; 95% CI, −1.52–0.99; *p* = 0.68; *I*^2^ = 63%). The ICU stay was examined in 10 trials with 1,459 patients. The lengths of ICU stay were less in the intervention group with a marginal statistical significance (Figure 5B; WMD, −0.22 days; 95% CI, −0.44 to −0.00; *p* = 0.05; *I*^2^ = 74%). The pooled analysis found no significant difference in hospital LOS between the groups (Figure 5C; WMD, −0.02 days; 95% CI, −0.73–0.70; *p* = 0.96; *I*^2^ = 88%).

**Figure 6:**
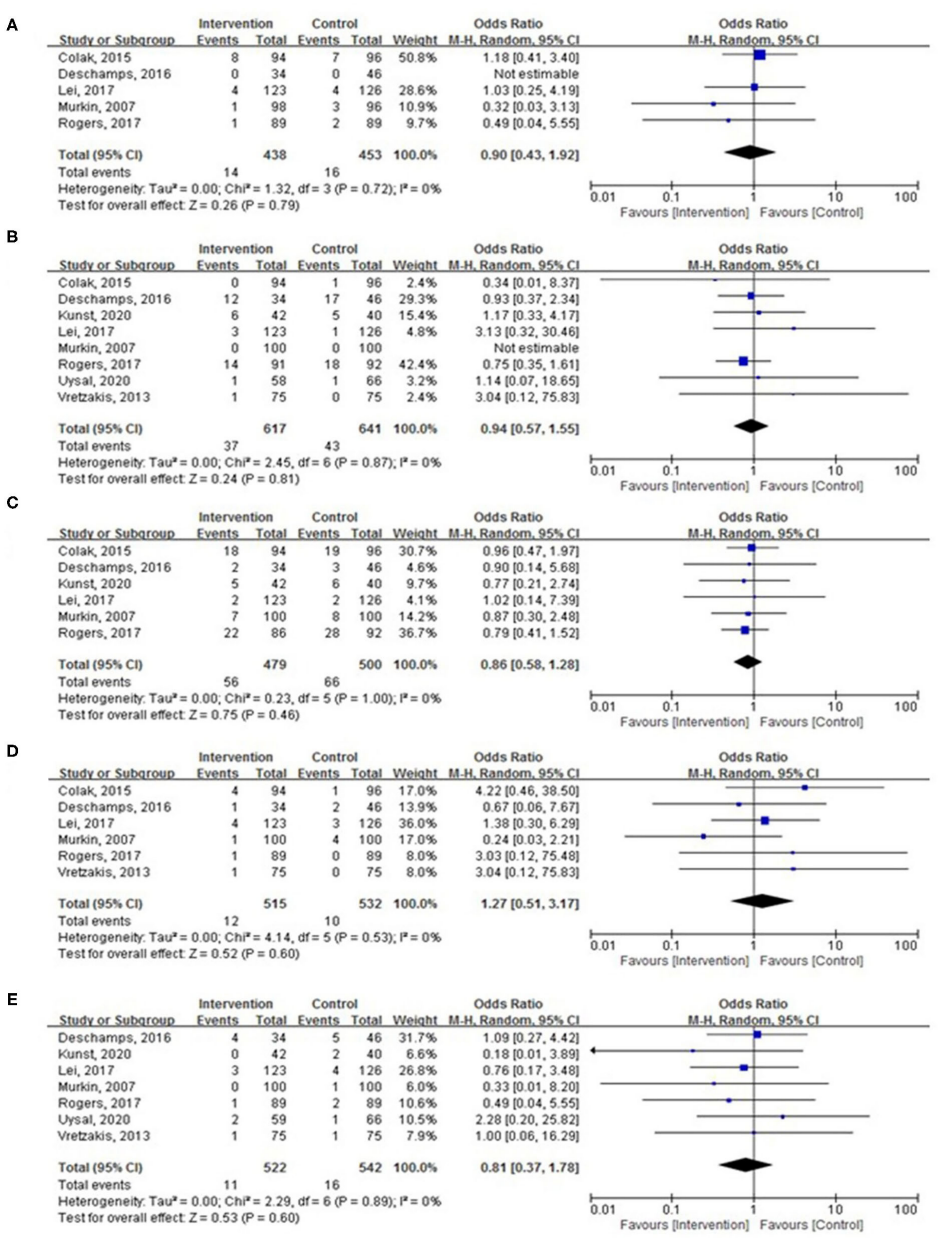
The pooled effect of cerebral oximetry monitoring-guided intervention on the incidence of **(A)** myocardial infarction (MI), **(B)** acute kidney injury (AKI), **(C)** surgical site infection, **(D)** cerebrovascular accident (CVA), and **(E)** hospital mortality.

There were no significant differences in the incidence of MI (Figure 6A; OR, 0.90; 95% CI, 0.43–1.92; *p* = 0.79; *I*^2^ = 0%), AKI (Figure 6B; OR, 0.94; 95% CI, 0.57–1.55; *p* = 0.81; *I*^2^ = 0%), surgical site infection (Figure 6C; OR, 0.86; 95% CI, 0.58–1.28; *p* = 0.46; *I*^2^ = 0%), CVA (Figure 6D; OR, 1.27; 95% CI, 0.51–3.17; *p* = 0.60; *I*^2^ = 0%), and hospital mortality (Figure 6E; OR, 0.81; 95% CI, 0.37–1.78; *p* = 0.60; *I*^2^ = 0%) between the two groups.

### Meta-Regression and Subgroup Analyses for the Potential Sources of Heterogeneity

Age, male, body mass index (BMI), previous MI, diabetes mellitus (DM), hypertension, CVA, chronic obstructive pulmonary disease (COPD), chronic renal failure, CPB duration, baseline left ventricular ejection fraction, a European system for cardiac operative risk evaluation, valve surgery, congenital heart disease (CHD) surgery, CABG surgery, and complex surgery were included in the random-effect univariate meta-regression analyses for POD, POCD, and ICU stay. The major sources of heterogeneity were age (coefficient = 0.09; *p* = 0.03; adjusted *R*^2^ = 0.99), BMI (coefficient = 0.53; *p* = 0.05; adjusted *R*^2^ = 0.97), and CHD (coefficient = −0.17; *p* = 0.02; adjusted *R*^2^ = 0.92) for POD, age (coefficient = 0.13; *p* = 0.04; adjusted *R*^2^ = 0.93) for POCD, DM (coefficient = 0.03; *p* = 0.07; adjusted *R*^2^ = 0.65), CVA (coefficient = 0.11; *p* = 0.02; adjusted *R*^2^ = 1), and COPD (coefficient = −0.10; *p* = 0.09; adjusted *R*^2^ = 1) for ICU stay as shown in Table 3.

**Table 3:** Meta-regression and subgroup analyses for the potential sources of heterogeneity.

**Variables**	**Endpoint**	**No. of comparisons**	**Coeff./OR/WMD WMD**	**95% CI**	***P-*value**		
**Univariate**			**Coeff**.				**Adjusted** ***R*^2^**
Age (years)	POD (Ln)	6	0.09	0.02 ~ 016	0.03		0.99
	POCD (Ln)	5	0.13	0.01 ~ 0.26	0.04		0.93
BMI	POD (Ln)	4	0.53	−0.01 ~ 1.07	0.05		0.97
CHD (%)	POD (Ln)	6	−0.17	−0.30 ~−0.04	0.02		0.92
DM (%)	ICU stay	7	0.03	−0.003 ~ 0.07	0.07		0.65
CVA (%)	ICU stay	5	0.11	0.04 ~ 0.18	0.02		1.00
COPD (%)	ICU stay	5	−0.10	−0.23 ~ 0.31	0.09		1.00
**Subgroup**						***I* ^2^**	***P*_Difference_** **value**
			**OR**				
1. Age (years)	POD	6					0.01
≥71.0		2	0.39	0.04 ~ 3.74	0.42	77.1%	
<71.0		4	0.22	0.05 ~ 1.00	0.05	81.50%	
2. Age (years)	POCD	5					0.001
≥63.0		2	0.89	0.55 ~ 1.44	0.64	0.00%	
<63.0		3	0.17	0.05 ~ 0.59	0.01	70.8%	
3.BMI	POD	4					<0.001
≥25.0		3	0.41	0.10 ~ 1.59	0.20	65.7%	
<25.0		1	0.02	0.004 ~ 0.09	<0.001	%	
4. CHD	POD	6					<0.001
≥20.0%		5	0.59	0.32 ~ 1.07	0.08	34.3%	
<20.0%		1	0.02	0.004 ~ 0.09	<0.001	%	
			***WMD***				
5. DM	ICU stay	7					<0.001
≥25.0%		5	−0.12	−0.41 ~ 0.17	0.42	56.7%	
<25.0%		2	−0.72	−0.98 ~−0.47	<0.001	0.0%	
6. CVA	ICU stay	5					<0.001
≥10.0%		3	−0.05	−0.30 ~ 0.19	0.66	41.4%	
<10.0%		2	−0.72	−0.95 ~−0.99	<0.001	0.0%	
7. COPD	ICU stay	5					0.06
≥16.0%		2	−0.56	−1.09 ~−0.04	0.04	0.0%	
<16.0%		3	−0.05	−0.30 ~−0.19	0.66	41.4%	

*POD, postoperative delirium; POCD, postoperative cognitive decline; ICU stay, intensive care unit stay; BMI, body mass index; CHD, congenital heart disease; DM, diabetes mellitus; CVA, cardiovascular accident; COPD, chronic obstructive pulmonary disease; Coeff., coefficient; OR, odds ratio; WMD, weighted mean difference; CI, confidence Interval*.

Subgroup analyses showed that studies with a mean age of <71.0 years old, a mean BMI of <25.0, and the proportion of CHD surgery being <20.0% had a low risk of POD than those with a mean age of ≥71.0 years old (OR: 0.22 vs. 0.39, *p* < 0.01 for a subgroup difference), a mean BMI of ≥25.0 (OR: 0.02 vs. 0.41, *p* < 0.001 for a subgroup difference), and the proportion of CHD surgery being ≥20.0% (OR: 0.02 vs. 0.59, *p* < 0.001 for a subgroup difference) as shown in Table 3.

Studies with a mean age of <63.0 years old had a lower risk of POCD than those with a mean age of ≥ 63.0 years old (OR: 0.17 vs. 0.89, *p* < 0.001 for a subgroup difference) as shown in Table 3.

Furthermore, patients undergoing the intervention of cerebral oxygen saturation with COPD without DM and CVA had a significantly reduced ICU stay than those without COPD (WMD: −0.56 vs. −0.05; *p* = 0.06 for a subgroup difference), with DM (WMD: −0.72 vs. −0.12; *p* < 0.001 for a subgroup difference), and CVA (WMD: −0.72 vs. −0.05; *p* < 0.001 for a subgroup difference) as shown in Table 3.

### Publication Bias Assessment and Sensitivity Analysis

Table 4 presents the results of publication bias. It was suggested that there was no obvious publication bias in POD (Egger's *p* = 0.06 and Begg's *p* = 0.06), POCD (Egger's *p* = 0.30 and Begg's *p* = 0.22), rSO_2_ desaturation (Egger's *p* = 0.22 and Begg's *p* = 0.33), and the length of ICU stay (Egger's *p* = 0.80 and Begg's *p* = 0.86).

**Table 4:** Evaluation of publication bias and sensitivity analysis.

**Index**	**OR (95% CI)**	***Z***	***P*-value**	***I*^2^ (%)**	***I*^2^'s *P***	**Egger's *P***	**Begg's *P***
POD	0.29 (0.10, 0.89)	2.16	0.03	81	<0.001	0.06	0.06
POCD	0.38 (0.16, 0.93)	2.13	0.03	78	=0.001	0.30	0.22
rSO_2_ desaturation	2.02 (1.25, 3.24)	2.89	0.004	81	<0.001	0.22	0.33
ICU stay	−0.22 (−0.44, 0.00)	1.93	0.05	74	<0.001	0.80	0.86

*OR, odd ratio; 95% CI, 95% confidence interval; POD, postoperative delirium; POCD, postoperative cognitive decline; rSO_2_, regional cerebral oxygen saturation; ICU, intensive care unit*.

### Trial Sequential Analysis

To confirm the pooled effect sizes of POD, POCD, or ICU stay as the true estimated effect, the required sample sizes for the POD effect are 2,415, the POCD effect is 3,115, and the ICU stay effect is 462. Future trials would need to include approximately further 1,468 POD event rates and 2,257 POCD event rates. However, the sample size of ICU stay (1,459 vs. 462) is enough for the estimated effect.

## Discussion

The present meta-analysis suggested that cerebral oximetry monitoring-guided intraoperative intervention was associated with a lower risk of POD and POCD and a shorter ICU stay in adults undergoing cardiac surgery. Similarly, we found an increased occurrence of POD with cerebral oximetry desaturation defined by both relative (below 70–90% baseline rSO_2_) or absolute (below 50–60% absolute rSO_2_) thresholds. These clinical benefits may be limited in patients with older age, diabetes status, high BMI, non-CHD, non-COPD, or a previous CVA.

In our study, the incidences of POD and POCD in 846 and 858 patients undergoing on-pump cardiac surgery were evaluated, of whom 149 (18.0%) and 284 (31.1%) were, respectively, diagnosed positive. POD is a common complication with a rate of 3.1–52% of adult patients undergoing cardiac surgery when defined as a disorder with an acute disturbance in attention and cognition (33). There are many factors associated with an increased risk of delirium, including advancing age, baseline cognitive impairment, preoperative comorbid conditions, and the type of surgery (34). Severe inflammatory responses, hypoperfusion and embolism related to CPB, are proposed to influence the cerebral oxygen supply/demand balance, and they cause subsequent delirium in a cardiac surgery patient (8). Greaves et al. did a meta-analysis to estimate the prevalence of cognitive impairments pre- and post-CABG, including delirium and dementia. They identified 215 studies with 91,829 patients and demonstrated that postoperative cognitive impairment increases to about 40% of patients acutely and then increases to nearly 40% in the long-term period (35). Their research findings provided important warnings about a cognitive decline in elderly patients, and more attention needs to be focused on the long-term (1–5 years) prognosis.

A multicenter observational study found that the prevalence of one or more rSO_2_ desaturations was 50–70% in cardiac surgery with CPB (36), prolonged rSO_2_ desaturation, and severe rSO_2_ desaturation significantly increased the risk of postoperative neurologic impairment (37, 38). In an observational study of 1,439 patients who underwent off-pump CABG surgery, intraoperative rSO_2_ reduction was associated with an increased risk of POD. The duration of rSO_2_ < 50% was 40% longer in patients with POD. For the prediction of POD, the cut-off value of intraoperative rSO_2_ was 50% for the total patient population, and 55% for patients younger than 68 years (39). Cournoyer et al. included 20 non-randomized studies in patients after cardiac arrest and concluded that a higher regional cerebral saturation is associated with improved resuscitation outcomes, especially the return to spontaneous circulation (40). In our meta-analysis, there is an increased occurrence of POD with cerebral oximetry desaturation defined by both relative (below 70–90% baseline rSO_2_) or absolute (below 50–60% absolute rSO_2_) thresholds. The results in this study are consistent with a previous report that suggested an association between cerebral oximetry desaturation with postoperative neurological impairment after cardiac surgery.

Determining whether the clinical benefits of rSO_2_ monitoring may interfere with patient characteristics in adult cardiac surgery has been an intriguing issue for a long time. Our analysis found that cerebral oximetry monitoring-guided intraoperative interventions had a low risk of POD in younger patients (<71.0 years old) and a lower BMI (<25.0), a significant reduction of POCD in patients with a mean age of <63.0 years old compared with control. Ding et al. conducted a meta-analysis and found that the intraoperative cerebral oxygenation monitoring could decrease the risk of POCD but have no effect on POD in non-cardiac and cardiac surgeries (41). Our meta-regression analysis showed that age was negatively correlated with the reduction in POD and POCD in the intervention group. In addition, we also found that the lengths of ICU stay were less in the intervention group with a marginal statistical significance. This result was similar to that reported by Zorrilla-Vaca et al., 1,300 patients from 9 RCTs in both cardiac and non-cardiac surgeries were analyzed with a higher heterogeneity (42). Subgroup analyses showed that patients with COPD, non-DM, and non-CVA had a significantly reduced ICU stay compared with the control. Cognitive dysfunction after cardiac surgery has been reported to be in association with an increased hospital stay, a prolonged ICU stay, a long-term cognitive dysfunction, and an increased risk for short-term mortality (2). Based on the findings from our study, determining whether pre-selecting a certain cohort of patients with a high risk of cognitive dysfunction may more likely show a significant decrease in the risks of POD and POCD by cerebral oximetry monitoring-guided interventions and eventually improve clinical outcomes, remains to be assessed in future, powered multicenter RCTs.

The major strength of our study includes gathering the largest sample size with only cardiac surgery, obtaining the positive effect of intervention based on cerebral SO_2_ monitoring on POD/POCD/ICU stay, and conducting a comprehensive exploration of clinically relevant influential factors (age, diabetes, BMI, and a previous CVA). However, this meta-analysis has several limitations. First, there are multiple mechanisms to develop POD and POCD, namely embolism, inflammation, and hypoperfusion, and they may not have the same risk factors. Hence, the potential interference of patient characteristics (age, diabetes, and peripheral vascular disease), cardiovascular medications, hemodynamic instability, and CPB duration may be underestimated. Second, the definitions for cerebral desaturation are different among the included trials. Third, non–English language publications were excluded and may result in a potential publication bias. However, the publication bias assessments were not obvious. Finally, the included research were all RCTs with comparatively small sample size, only 7 RCTs qualified for the meta-regression analysis, and therefore, the conclusions may not be robust but hypothesis generating. To clarify the neuroprotection effectiveness of intraoperative interventions followed by optimizing cerebral oxygenation in cardiac surgical patients, further large randomized trials are needed.

In conclusion, the available evidence in the meta-analysis suggests that cerebral oximetry monitoring-guided intraoperative interventions are correlated with a lower risk of postoperative cognitive dysfunction and a shorter ICU stay in adults undergoing cardiac surgery. These clinical benefits may be limited in patients with older age, diabetes status, high BMI, non-CHD, non-COPD, or a previous cardiovascular accident. Based on the fact that an intraoperative intervention followed cerebral oxygenation may provide significant benefits in patients with a high-risk status, and further trials are needed to verify the effectiveness of cerebral oxygen saturation intervention threshold settings in improving cognitive function.

## Data Availability Statement

The original contributions presented in the study are included in the article/supplementary material, further inquiries can be directed to the corresponding author/s.

## Author Contributions

L-JT and C-HZ made a substantial contribution to the conception and design of the work and manuscript drafting. L-JT, SY, C-HZ, and F-XY contributed to the acquisition, analysis, and interpretation of the data. All authors were involved in drafting and revision of the manuscript for important intellectual content and approved the final version to be published.

## Conflict of Interest

The authors declare that the research was conducted in the absence of any commercial or financial relationships that could be construed as a potential conflict of interest.

## Publisher's Note

All claims expressed in this article are solely those of the authors and do not necessarily represent those of their affiliated organizations, or those of the publisher, the editors and the reviewers. Any product that may be evaluated in this article, or claim that may be made by its manufacturer, is not guaranteed or endorsed by the publisher.
